# Supplementary figures and images for: The delayed clearance of Talaromyces marneffei in blood culture may be associated with higher MIC of voriconazole after antifungal therapy among AIDS patients with talaromycosis
Source: PLoS Negl Trop Dis. 2023 Apr 3;17(4):e0011201. doi: 10.1371/journal.pntd.0011201 (PMC10101635; doi:10.1371/journal.pntd.0011201)

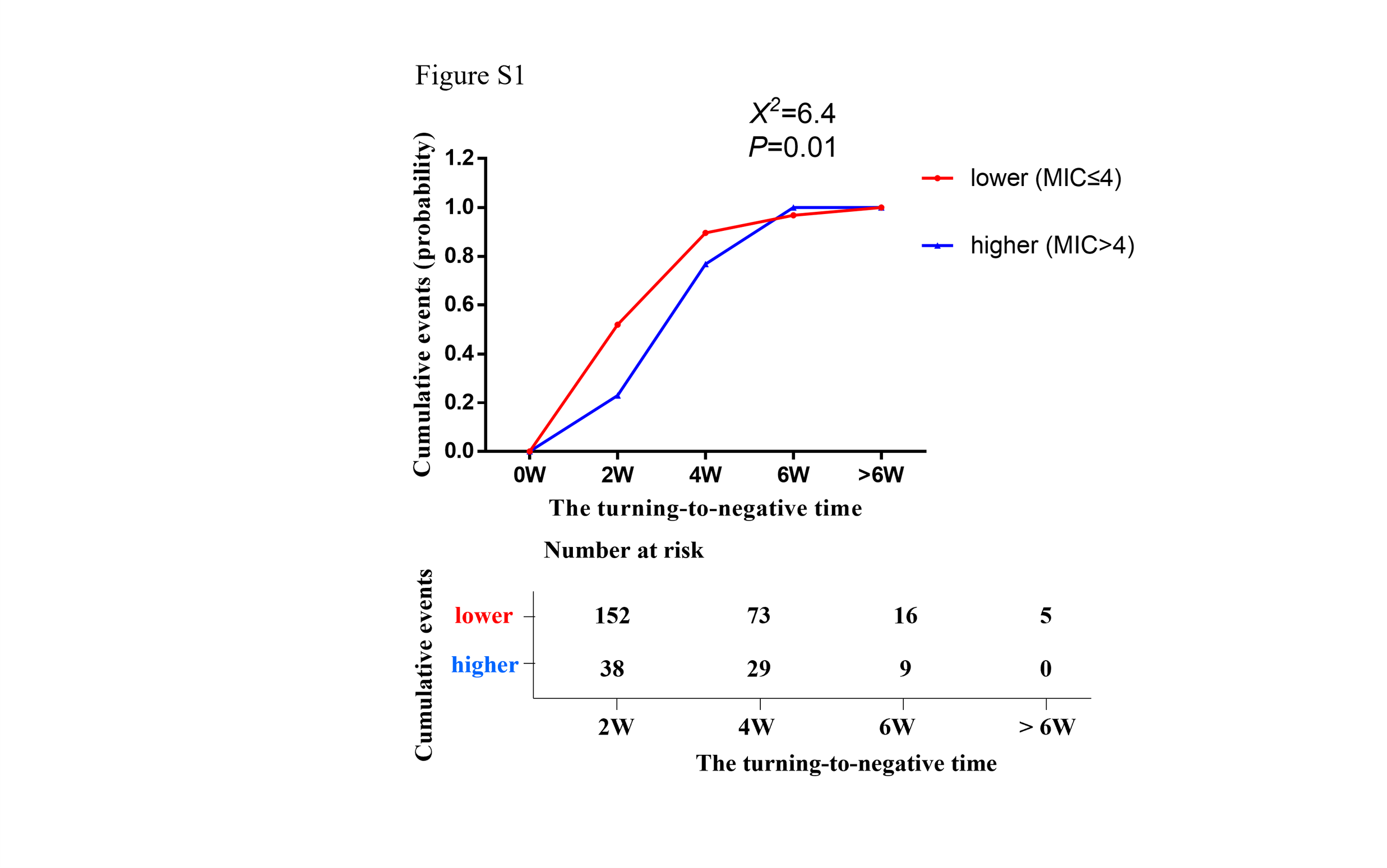

Supplement: S1 Fig — Strains from 190 patients were plit into two groups according to the MIC values for fluconazole in vitro: lower (MIC≤4) and higher (MIC>4). Cumulative negative conversion rate of blood cultured fungi was assessed between the two groups after 2, 4, 6 weeks and more than 6 weeks of antifungal treatment. P<0.05 is considered statistically significant. (TIF) [file pntd.0011201.s001.tif]
