# Supplementary material for: The delayed clearance of Talaromyces marneffei in blood culture may be associated with higher MIC of voriconazole after antifungal therapy among AIDS patients with talaromycosis
Source: PLoS Negl Trop Dis. 2023 Apr 3;17(4):e0011201. doi: 10.1371/journal.pntd.0011201 (PMC10101635; doi:10.1371/journal.pntd.0011201)
Supplement: Attachment — Submitted filename: 20220422 FGB-D-22-00032_Reviewercomment_MN.docx [file pntd.0011201.s003.docx]

**Reviewer comments:**

**Summary:**

In the study of Aspergillus fumigatus, a critical human pathogen, the authors demonstrated a search of genomic locus that encoded a mutated transposon, with a mutated and inactive transposase, aft4 through bioinformatic analysis. The authors further deleted the whole region and found it didn’t affect the general vegetative growth phenotypes and stress tolerance to pH, heat and iron, or virulence in animal models. Replacement of the locus with a fluorescent protein Venus under an iron-responsive promoter led to visible fluorescence. However, the expression of the downstream gene seemed to be affected by knocking in of the Venus dependent on the construct. This was not further investigated. Further analysis of *A. fumigatus* genomes identified presence of this locus within *A. fumigatus* strains, leading to speculation that the non-sense mutation arose in the species early during evolution. Overall, the authors clearly demonstrated presence of this locus and its potential use in genetic engineering. Yet it is worth discussing whether the inactive transposons could be identified across species and genera and the potential of this approach in identifying genomic safe-haven loci in other industrially valuable fungi.

Some minor comments for consideration are listed below:

**Results:**

Fig. 4a: the fluorescent images were faint to visualize the fluorescence

Fig. 4b: The hyg-aft4 transformant seemed to have stronger YFP signal. Was this transformant the only one confirmed to have single integration by southern analysis (Supplementary figure 3)? Are there heterogeneity in *venus* expression level among confirmed engineered fluorescent strains?

Figure 4: Even though is clear that exogenous gene can be expressed while inserted in the aft4 locus, it is not shown if high-level gene expression can be achieved. It would be more informative if relative gene expression can be shown with a control, such as beta-actin, gpdA or hapX genes, to demonstrate its use as a landing pad for engineering.

Supplementary fig 4: lacks WT and construct controls

Page 20, line 11: It is an interesting observation. To confirm this is dependent on the construct type, multiple confirmed transformants should be evaluated for expression of venus, down- and up-stream *orf*.

Page 21, line 1: please include an alignment of typical NH1, NH2 and NH3 aft4 locus to show the difference among the three orthologous regions.

**Discussion:**

Page 22, Line 20: Is the aft4 locus in D141 syntenic with CEA10 and Af293?
