# Supplementary material for: The delayed clearance of Talaromyces marneffei in blood culture may be associated with higher MIC of voriconazole after antifungal therapy among AIDS patients with talaromycosis
Source: PLoS Negl Trop Dis. 2023 Apr 3;17(4):e0011201. doi: 10.1371/journal.pntd.0011201 (PMC10101635; doi:10.1371/journal.pntd.0011201)
Supplement: Attachment — Submitted filename: response to reviewer(3).docx [file pntd.0011201.s004.docx]

Dear Editors and Reviewers,

*PLOS Neglected Tropical Diseases*

Thank you for your comments on the revision. We have provided explanations where necessary. Please, find below the point-by-point responses to the reviewers' comments and the attached revised version of the manuscript.

Sincerely,

Linghua Li

Reviewer #1:

Comment 1: Some results lack clear description, presentation and interpretation.

Response 1: Thank you very much for your proposal. We have described some results again to make them easier to interpret. Specific modifications have been marked in the manuscript.

Comment 2: Please check grammatical errors and revise figures and tables.

Response 2: Thanks for your suggestions. The grammas have been checked and figures and tables have been revised.

Reviewer #2

Comments  
Figure 1: correct the black background
Figure 2: increase the quality or make a diagram. If possible discard the figure as they do not provide much relevant content.

Response:

Figure 1: Thanks for your suggestion. We have submitted a correct figure.

Figure 2: Thanks for your suggestion. We have removed it in the revised manuscript.

Reviewer #3:

Comment 1: The MIC values of voriconazole given on the Table 1 for the both positive and Negative groups are within the reported MIC90 values of voriconazole, therefore, it is presumable that the isolates are susceptible to voriconazole and the observed difference is a mere variation among the population and cannot be attributed to resistant phenotype. The authors should consider redoing the statistical analysis with the percentage of strains that has MICs above the MIC90 value. The authors are recommended to do a similar analysis on fluconazole using MIC90.

Response 1: Thanks for your suggestion. The analysis of strains with MIC larger than MIC90 will confirm the conclusion, as you suggested. However, currently, all *T. marneffei* strains in our hospital are sensitive strains with relatively low MIC values. Our study also conforms to the current literatures that the MIC values of *T. marneffei* strains to voriconazole in vitro are less than 0.06µg/mL (1-3). Therefore, we are unable to differential the strains higher or lower than MIC90.

In order to confirm our conclusion, we split 190 strains according to the MIC values of voriconazole into: higher (MIC≤0.008), medium (0.008<VOA≤0.015), and lower (VOA>0.015) groups and performed cumulative analysis to assess negative conversion rate of blood cultured fungi. Patients in the higher group, medium group, and lower group were 84, 85, and 21, respectively. The cumulative negative conversion rate of blood cultured fungi among three groups after 2, 4, 6 weeks and more than 6 weeks of antifungal treatment was significantly different, indicating that the MIC values of voriconazole significantly affects the clearance of *T. marneffei* in blood. Similarly, we found the same performance in fluconazole. The results have been uploaded as Figure 2 and supplementary Figure S1. Please check Figure 2 and Figure S1 listed below and in the manuscript.

Figure 2: The MIC values of voriconazole might affect the clearance of *T. marneffei* in blood cultures among AIDS patients with talaromycosis after antifungal therapy. Strains from 190 patients were split into three groups according to the MIC values for voriconazole *in vitro*: higher (MIC ≤ 0.008), medium (0.008 < VOA ≤ 0.015), and lower (VOA > 0.015). Cumulative negative conversion rate of blood cultured fungi was assessed among three groups after 2, 4, 6 weeks and more than 6 weeks of antifungal treatment. *P*<0.05 is considered statistically significant.

Figure S1: The MIC values of fluconazole might affect the clearance of *T. marneffei* in blood cultures among AIDS patients with talaromycosis after antifungal therapy. Strains from 190 patients were split into two groups according to the MIC values for fluconazole in vitro: higher (MIC≤4) and lower (VOA>4). Cumulative negative conversion rate of blood cultured fungi was assessed between the two groups after 2, 4, 6 weeks and more than 6 weeks of antifungal treatment. *P*<0.05 is considered statistically significant.

Comment 2： Table 1 and Page 6(lines 139-140). The authors states that there were no significant differences were evident between the groups with respect to co-infections. It appears that authors looked for plausible correlation between a co-infection and delayed clearance of T. marneffei. Did the authors do the analysis with individual co-infection agent? The authors are suggested to do the analysis with individual co-infection agent to see any potential correlation exists.

Response 2: We appreciated your suggestion. It is true that coinfections can affect the clearance of *T. marneffei* from the bloodstream, and we have added data about co-infected tuberculosis (TB) and pneumocystis pneumonia (PCP) in Table 1. TB and PCP levels were not significantly different between the two groups, ruling out the possibility of coexisting opportunistic infections affecting fungal clearance from blood.

Comment 3：The authors have used AST as marker, however increased AST could be due to several reasons. The authors have put little effort to explain the observed co-relation. Some of the co-infection agents could enhance the AST level. Liver is one of the major organs where T. marneffei infects, it is plausible that the extensive liver damage caused by a co-infective agent such as hepatitis B, delayed the clearance of fungi.  Also, any kind of chemotherapeutic agents that the patient received could also influence the level of AST.

Response 3：Thanks for your suggestion. In the Table 1, we added the data regarding coinfection with HBV and HCV of the two groups, and the co-infection rate between the two groups was not significantly different after chi-square analysis. Additionally, we compared co-sulfamethoxazole, cefalexin and other concomitant medications, and no significant differences were observed.

References

1.Liu D, Liang L, Chen J. In vitro antifungal drug susceptibilities of Penicillium marneffei from China. J Infect Chemother 2013;19:776–778. doi: 10.1007/s10156-012-0511-7.

2. Lau SK, Lo GC, Lam CS, Chow WN, Ngan AH, Wu AK, et al. In vitro activity of posaconazole against Talaromyces marneffei by broth microdilution and etest methods and comparison to itraconazole, voriconazole, and anidulafungin. Antimicrob Agents Chemother 2017;61:e01480–e1516. doi: 10.1128/ AAC.01480-16.

3. Zhang J, Liu H, Xi L, Chang YC, Kwon-Chung KJ, Seyedmousavi S. Antifungal susceptibility profiles of olorofim (formerly F901318) and currently available systemic antifungals against mold and yeast phases of Talaromyces marneffei. Antimicrob Agents Chemother 2021;65:e00256–e321. doi: 10.1128/AAC.00256-21.
